# Role of household waste, governance quality, and greener energy for public health: Evidence from developed economies

**DOI:** 10.3389/fpubh.2022.1005060

**Published:** 2022-10-21

**Authors:** Jiping Wei, Lihua Xu, Jing Zhou

**Affiliations:** ^1^School of Management, Chengdu University of Traditional Chinese Medicine, Chengdu, China; ^2^Human Resource Department, Chengdu University of Traditional Chinese Medicine, Chengdu, China

**Keywords:** public health, household waste, governance quality, renewable energy, urbanization, economic growth, method of moment quantile regression

## Abstract

In the current times, the global economies and international organizations declared that pollution is one of the prominent causes of declined human health. Still, most literature is biased toward economic sustainability and ignores such vital issues. The current study tends to identify the factors affecting public health in the Group of Seven economies except for Italy (G6). Specifically, this study aims to investigate the influence of household waste (HHW), bureaucratic quality (BQ), democratic accountability (DA), urbanization growth (URP), GDP per capita, and renewable energy use (EPR) on public health, throughout 1996-2020. This study uses advanced panel data approaches and finds the heterogeneity of slope coefficients, the dependence of cross-sections, and the persistence of cointegration between the variables. The asymmetric distribution of data leads to employing the novel method of moment quantile regression. The estimated results reveal that URP, GDPPC, and EPR significantly increase domestic general government health expenditures, improving public health. However, HHW and BQ adversely affect public health by reducing health expenditures. The robustness of the results is tested *via* utilizing the panel quantile regression. Based on the empirical findings, this study suggests policies regarding the improvement in public health expenditure, R&D investment, spending in renewable energy sector, and strengthening of the institutional quality.

## Introduction

The majority of the people around the world live below the poverty line and globalization has complicated the health equality challenge in the world (1). Since the COVID-19 outbreak, health expenses have further augmented around the world affecting the rich and poor nations more. The availability of healthcare facilities for all is on the Sustainable development goals (SDG) agenda; however, providing primary health facilities to people has become a question mark since the pandemic. The health expenses in tackling health risks differ for different economies depending on their financial stability (2). Some countries spent < 1% of their financial budget, while some spent a significant share of their GDP on healthcare. Several factors are responsible to influence healthcare expenditures such as governance, accountability, civil society, and economic policies (3). The pandemic has devastatingly influenced our lives across the globe, especially when a person needs healthcare services. International organizations and developed economies are adopting strategies for limiting environmental pollution and overcoming significant challenges like health inequality. The world is facing several global economic, environmental, and social challenges and the developed economies are standing at a crossroads. For that reason, sustainability either health or the environment has now become a global concern. To secure world order and democracy, the Group of Seven economies must come forward to ensure health sustainability because these countries have a substantial role in restructuring international policies and supporting vulnerable poor economies (4). The deteriorating health risks affect sustainability and economic development. Therefore, the increasing health expenditure by the government positively impacts the public's health and ultimately encourages sustainable development (3). Further, conferring at the G7 summit (5), developed economies broadly discuss issues like climate change, economic sustainability, and global health besides providing an influential strategic role. Moreover, G7 has worked on countless health crises and health commitments for sustainability. Recently they joined the session on global health concerns to reinforce strategies for health emergencies (5, 6).

In prevailing literature elaborates on the connections of study variables concerning health. In the existing literature, the association between household waste, Bureaucratic Quality, Democratic Accountability, and health expenditures is not a highly discussed area among scholars. Though, some studies documented the connotation regarding health outcomes. Nazarov and Obydenkova (7) examined that Democratic accountability is linked to better choices and policies for the public and health and is an integral part of good governance. The efficiency is democratic accountability is significant for public health in the economy. Likewise, Bureaucratic Quality improves health quality if appropriately managed (3). In an innovative study, Gutberlet and Uddin (8) evaluated household waste as not usually toxic but harmful to public health because it spreads hazardous diseases to the general public. The literature on health expenditure is limited and fixated on economic growth and renewable energy consumption. The relationship between renewable energy and economic growth on health expenses is momentous (9–12). Hence the present study is interested in investigating the role of household waste and governance in public health expenditures.

Attributable to the research requirements, the study aims to investigate the following three objectives. First, the role of governance is examined in public health in G7 countries, except Italy. The Bureaucratic Quality and Democratic Accountability proxy for governance quality and government health expenditures are employed in two modifications alongside explanatory variables. Second, the impact of renewable energy on public health expenditures is observed in both econometric models. The study uses novel variable electricity produced from renewable sources to accomplish this objective. Third, the study examines household waste's influence on health expenditures in G6 economies in both econometric models.

As *per se*, governance, renewable energy, and household waste significantly impact public health in an economy. Thus, the study is motivated to assess bureaucratic quality, democratic quality, household waste, and green energy on public health expenditures in G6 countries. Since the Sustainable development program 2030, the global environmental crisis and health disparity have increased. The developed nations have confronted several challenges in the past and now it is high time that G7 economies come to the forefront to tackle challenges like health inequality, poverty, and hunger to make themselves an example for other nations worldwide (4). Additionally, these developed economies have a major role in shaping global policies for sustainability. The empirical results have demonstrated significant determinants of government health expenditures. Institutional quality is significant in evaluating the health expenditures for improving health quality. Hence, the study significantly examines the role of governance (BQ and DA) and clean energy on public health for G6 nations to provide substantial outcomes in evaluating policies for mitigating these chief challenges like health predicament.

The study contributes to the literature in threefold subsequent ways.

First, the study's originality is that it is foremost in evaluating the role of governance quality on public health expenditures. After the pandemic, the world's health concerns escalated, prompting countries and governments to adopt strict measures to ensure health quality. Therefore, the study evaluates the governance role in G6 nations (dropping Italy from the G7 list due to data unavailability). For this, the influence of institutional factors like Bureaucratic Quality (BQ) and Democratic Accountability (DA) on health expenditures are scrutinized and considered new input in the extant literature because they substantially affect public health outcomes (3). Moreover, the study contributes to the prevalent literature by examining the role of institutional quality in two different econometric models. The justification for selecting two different models is that taking both the institutional (Bureaucratic Quality and Democratic Accountability) variables in one model could lead to estimation bias or creates the question of multi-collinearity. Therefore, the study scrutinizes the role of the said novel variables like (BQ and DA) on government health expenditures that will support analyzing the governance quality over public health. The current findings meaningfully provide new evidence to the empirical literature.

Second, attributable to panel data concerns and the issue of non-linearity, the objectives are accomplished by employing modern and updated panel econometric analysis. The uniqueness of the study in the prevailing literature lies in employing Novel approaches such as MMQR that provide specified results besides verifying the long-run linkage among variables. The non-parametric Quantile regression is also used to assess the influence of each explanatory variable on DGHE providing evidence of the validity of the research models. Further, the present work employs the pairwise panel Granger causality tests at the end which are more effective at addressing the problems with panel data. Supplementary details are elaborated in section Data and methods of the manuscript correspondingly.

Third, the study utilizes an updated data period from 1996 to 2020. In addition, this study scrutinizes the association between Bureaucratic Quality and Democratic Accountability, Economic Growth, Renewable Energy use, Household waste, and Urban population on Public Health in two different modifications in a panel study for the Group of six developed nations by practically contributing to the first time in the prevailing literature.

The rest of the manuscript is organized as follows. The next Section Literature review is on the review of the empirical literature: Sections Data and methods and Results and discussions document methodology and results with discussions, respectively. Lastly, the conclusion and policy implications are mentioned in Section Conclusion and policy implications of the study.

## Literature review

The empirical literature review on variables under consideration is elaborated in this section of the manuscript for clarification.

### Renewable energy use, household waste, urban population, and public health

The relationship between health expenses and economic growth is inverse. Alhassan et al. (13) examined the relationship between health expenditures and economic growth. The empirical results demonstrated an inverse association between health expenses and economic growth. Ghorashi and Rad (14) examined the unidirectional causal association between economic growth and health expenditures in Iran. However, the causality runs from health expenses to economic growth. In the case of Turkey, the cointegration analysis demonstrated a unidirectional and significant association between health expenses and economic growth (9). In Asian economies, Nasreen (10) observed positive associations between economic growth and environmental pollution on health expenditures. The findings depicted that the magnitude of the coefficient of environmental pollution is higher than economic growth and bidirectional causality exists between economic growth and health expenses. Similarly, a bi-directional relationship between health expenses and economic growth in China was demonstrated (15). In contrast, Kutlu and Örün (16) examined a panel econometric analysis in OECD economies. The empirical findings discovered a long-run positive relationship between GDP growth and health expenditures because increasing economic activities increase pollution, thereby increasing health expenses. Likewise, a positive linkage is observed between economic growth and health expenses (17). Additionally, (18) observed an asymmetric (positive/negative) association between economic growth and health expenditure due to different levels (high/low) of human capital.

The increased renewable energy consumption is inversely related to carbon emissions leading to lesser health effects. Therefore, the more renewable energy is consumed lesser will be the health expenses. Ullah et al. (19) examined the association between renewable energy and health expenditures. The empirical findings depicted a negative association between them. Further, a bi-directional causal association occurred in the case study of G7 economies. The empirical findings suggest that health expense impacts the renewable energy budgets in the country (12). In another novel study, Shahzad et al. (20) explored the negative impact of renewable energy on health expenditures and carbon dioxide emissions. The granger analysis demonstrated a unidirectional causal association between renewable energy consumption and health expenditures. Mehmood et al. (11) elucidated that increasing renewable energy consumption positively impacts environmental quality, reducing health risks and expenditures on health. They presented a negative association between renewable energy and health expenses by employing FMOLS and DOLS econometric analysis for investigating long-run relationships. In the study of EU nations, Sasmaz et al. (21) observed that improving renewable energy aids public health by upsurging health spending in European countries. The study demonstrated a unidirectional association between renewable energy and health expenditures in countries that joined before the year 2000, while a two-way directional association occurred in countries that joined after the year 2000.

The increased health risks increase public and private health expenses in the economy. According to World Health Organization (22), millions of people are affected by household air pollution and prematurely die from household pollution and waste due to increasing health concerns and environmental deterioration (23). In the existing literature, the association between household waste and health expenses is not a highly discussed area in academics. However, a few studies examined aspects related to the variables. Zeeshan et al. (24) scrutinized that environmental waste positively affects health expenditures. The increasing waste significantly raises health spending because of increased health risks. (25) inspected the factors of household waste disposal that impact health expenses. The study revealed significant factors that influence expenditures. The areas having no household waste facilities are suffering from health risks.

The increasing industrialization significantly increases urbanization across the globe. Thus, urban growth has a substantial impact on the expenditures on health in an economy. Ahmad et al. (15) examined the bi-directional causal association between urban growth and health expenditures in China. The findings exhibited a positive association between them. For that reason, urbanization significantly increases health expenditures. Çetin and Bakirtaş (26) observed that urban growth caused an increase in health spending. The econometric analysis confirmed the association. Moreover, Fattahi (27) discovered the reinforcing role of urbanization in escalating health expenditures. Recently, Shao et al. (28) analyzed the positive and significant association between urbanization and health expenses. The findings depicted that increasing urban growth leads to increased expenditures on health and behavior (29). In addition, Ahmad et al. (30) discovered the asymmetric relationship between urban growth and health spending.

### Role of bureaucratic quality and democratic accountability on public health

Literature is scarce on the relationship between democratic responsibility on public health, though the following set of studies from the extant body of knowledge aid in elucidating the aspects of the connection. (31) stated that weak accountability is directly proportional to weak health systems leading to decreasing government health expenses in an economy. Farag et al. (32) suggested and highlighted the importance of government health spending and democratic effectiveness using the fixed effect model. The results demonstrated that the improvement in democratic accountability increases the efficiency of health expenditures in an economy. Another study inspected mixed findings on the relationship between governance and health expenditures (33). Also, Liang and Mirelman (34) scrutinized the linkage between government health spending and democratic accountability. The empirical findings depicted the positive and diminishing impact of democratic accountability on health expenditures when the economy and government are stable.

Heaton and Walid (35) recommended that for rampant healthcare systems, the efficient quality of bureaucracy is required. In a novel article, Segel (36) states that bureaucratic quality is one of the determinants of enhanced healthcare systems in the USA. The empirical research by Makuta and O'Hare (37) determined that good governance, a proxy for bureaucratic quality, aids in reducing mortality rates due to levitating the government health expenditures in the country. Rehmat et al. (38) also examined the significant relationship between bureaucratic quality, democratic accountability, and health outcomes.

To the best of the authors' knowledge, the existing literature hasn't focused on scrutinizing the impact of household waste, Bureaucratic Quality, and Democratic Accountability on public health, especially on health expenditures. Therefore, the present study aims to analyze the stated linkage in G6 economies by providing additional evidence in the empirical literature.

## Data and methods

### Data and model specification

Based on the prevailing literature, this study observed the need for an empirical study regarding human health, influenced by numerous economic, governance, institutional, environmental, and energy-related factors. For omstance, it ican be observed from the above mentioned literature that the pollution level and wastes are playing a substantial role in human health declination (16, 39). As a matter of fact, the G6 economies are still using carbon intensive energy resources, which although provides higher economic, along with the increase in public heath expenditure (10). Thus, it can be observed that both the wastes and economic growth could have a role in the health expanditures, which is relevant to study empirically. Additionally, this study observed that the recent trend in renewable energy consumption is rapidly increasing, which could have also have a substantial influence on the public health as renewables are the an alternative source of the traditional fossil fuel (11), therefore, the importance of renewable energy could not be ignored in the public heath related expenditures. Besides, the bureaucratic quality and democratic accountability affects the economic and political stability in the country. Therefore, the importance of these variables cannot be ignored in the public health spendings in the developed economies as these variables have a substantial influence over the gadgetry decision of the government (31, 32).

In this context of above-mentioned discussion, the current study proxied domestic general health expenditures (DGHE) for public health [measured as domestic general government health expenditure per capita (current US$)]. On the other hand, this study uses household waste (HHW), bureaucratic quality (BQ), democratic accountability (DA), urbanization growth (URP: urban population), gross domestic product (GDPPC: constant US$ 2015), and renewable energy [EPR: Electricity production from renewable sources, excluding hydroelectric (kWh)]. Following the study of Wei et al. (39), this study constructed the following models:

### Model-1


(1) $$\begin{array}{l} DGHE_{it}=\beta_{0}+\alpha_{1}HHW_{it}+\alpha_{2}BQ_{it}+\alpha_{3}URP_{it}\\ \;+\alpha_{3}GDPPC_{it}+\alpha_{3}EPR_{it}+\varepsilon_{it}, \end{array}$$

Where Model-1 indicates that HHW, BQ, URP, GDPPC, and EPR combinedly are the function of DGHE.

### Model-2


(2) $$\begin{array}{l} DGHE_{it}=\beta_{0}+\alpha_{1}HHW_{it}+\alpha_{2}DA_{it}+\alpha_{3}URP_{it}\\ \;+\alpha_{3}GDPPC_{it}+\alpha_{3}EPR_{it}+\varepsilon_{it}, \end{array}$$

Where Model-2 indicates that HHW, DA, URP, GDPPC, and EPR combinedly is the function of DGHE, further, β and α′*s* represent intercept of the model, and slopes for each variable, respectively. The subscript “*i*” and “*t*” reports the cross-sections and time series, respectively, which are the G6 countries, including the United States, the United Kingdom, Japan, Germany, France, and Canada, covering the period from 1996 to 2020. The reason for dropping Italy from the G7 list is the data unavailability. Whereas, justification for selecting two models is that taking both the institutional (BQ and DA) variables in one variable could lead to estimation bias or the issue of multi-collinearity. Moreover, the random error of both models is captured *via* ε. Data for all the variables are extracted from the World Development Indicators of the World Bank (40)^1^.

### Estimation strategy

This study investigates descriptive statistics for researched elements to provide a comprehensive overview of panel data. In particular, descriptive analytics covers the mean, median, and range values, the latter containing the minimal and maximum observations of data. This research also investigates the variable's standard deviation, which demonstrates the temporal variable's volatility by demonstrating the data's dispersion from the mean value of a particular variable. Further, two normality metrics are performed to analyze the data's distributive properties: skewness and Kurtosis are used to verify whether a variable's distribution meets the normalcy criteria. Nonetheless, Skewness and Kurtosis provide genuine information on the variable's dispersion. However, this approach tackles the issue of normality with more precision. This study used the Jarque and Bera (41) normality test, which assesses skewness and excess Kurtosis and maintains them equal to zero as a null hypothesis for normal distribution. The following is Jarque-Bera's mathematical equation for normality statistics:


(3) $$\begin{array}{l} JB=N.\frac{1}{6}\left(S^{2}+\frac{{\left(K-3\right)}^{\;2}}{4}\right)., \end{array}$$

Since this research focuses on panel data, panel data techniques are feasible to employ. The first phase of this panel inquiry is to evaluate the Slope heterogeneity and Cross-section Dependence of the selected Panel data. In certain industries, panelist nations may have both similarities and distinctions. However, the similar characteristics of countries may lead to inaccurate forecasts in econometric research, particularly in panel estimations (39, 42). Therefore, assessing if the G6 economies have similar or distinct characteristics is essential. In this instance, the slope coefficient homogeneity (SCH) test designed by Pesaran and Yamagata (43) is used to examine coefficients that were comparable to the hypotheses: “slope coefficients are homogeneous.” The basic formulas for the specification described above are as follows:


(4) $${\hat{\Delta }}_{SCH}=\;{\left(N\right)}^{1/2}{\left(2k\right)}^{-1/2}\;\left(\frac{1}{N}Ś-K\right).,$$

(5) $${\hat{\Delta }}_{ASCH}=\;{\left(N\right)}^{1/2}\;{\left(\frac{2K(T-K-1)}{T+1}\right)}^{-1/2}\;\left(\frac{1}{N}Ś-2K\right).,$$

Where ${\hat{\Delta }}_{SCH}$ symbolizes the slope coefficient homogeneity (SCH) and ${\hat{\Delta }}_{ASCH}$ denotes the slope coefficient homogeneity (SCH) after adjustment.

In today's globalized society, several variables may increase a nation's dependence on the rest of the world, such that an alteration in a variable in one country may have repercussions in another country or area. However, ignoring cross-sectional dependence may also lead to erroneous and confusing findings (39, 44). Therefore, we used Pesaran (45) cross-section dependence (CD) test to assess cross-section reliance across the G6 countries. The following is an overview of the test described above, which takes cross-sectional independence as the null hypothesis.


(6) $$\begin{array}{l} {\text{CD}}_{Test}=\sqrt{\frac{2T}{N\left(N-1\right)}}\sum_{i=1}^{N-1}\sum_{k=1+i}^{N}T_{ik.}, \end{array}$$

Due to the predominance of panel data concerns, i.e., SCH and CD, a suitable unit root estimation method is used to tackle these problems. This research used the cross-sectional IPS (i.e., CIPS) test devised by Pesaran (46), which is more robust than the ADF, Levin, Len, and Chu, etc., unit root tests in compensating for the panel data problem and producing more reliable findings. Pesaran (47) first presented a factor modeling method to assess unexplained cross-sectional means for cross-sectional dependence. Pesaran (46) integrates the mean and first differentiated cross-section lags into the ADF linear regression using the same procedures. This method permits cross-sectional dependency despite the imbalance of the panel (N > T or T > N). By using the following equation, the CIPS empirical results might be obtained:


(7) $$\begin{array}{l} CIPS=\;N^{-1}\;\sum_{i=1}^{N}CADF_{i.}, \end{array}$$

The Pesaran (46) CIPS tests assume the existence of a unit root in a panel time series.

Since the previous estimator discloses the stationary properties of each variable, it is necessary to determine if they have a long-term equilibrium connection. This research employs two-panel cointegration methods: the cointegration test of Kao (48) and the cointegration test of Pedroni (45). In these tests, Kao (48) assesses the Dickey-Fuller t, Modified Dickey-Fuller t, the Augmented Dickey-Fuller t, the unadjusted Dickey-Fuller t, and the unadjusted Modified Dickey-Fuller t, while the Pedroni (45) evaluates the modified Phillips-Perron t, the Phillips-Perron t, and augmented Dickey-Fuller t statistics. Both of these tests presume that the examined variables have no long-term link. Nonetheless, suppose the empirical findings of these specifications are significant at any 10, 5, or 1% levels. In that case, the null assumption will be rejected, and it will be predicted that cointegration exists between the variables.

Since the investigated variables displayed stationarity, one of the conditions for determining long-run elasticities, and also had the characteristics of long-run cointegration. Therefore, the long-run elasticities may be derived. Consequently, the present research considers the asymmetrical data distribution, demanding using a novel Method of Moment Quantile Regression (MMQR) technique. Koenker and Bassett Jr. (49) presented the quantile regression method to assess the mean dependency and conditional variance for minimizing non-linearity issues. Machado and Silva (50) developed the MMQR technique for assessing the distribution of quantile estimates based on this methodology (51). The sophisticated formula for the conditional location-scale variation *Q*_*y*_(τ|*R*) is as follows:


(8) $$\begin{array}{l} Y_{it}=\;\alpha_{i}+\beta R_{it}+\;\left(\gamma_{i}+\rho {Ź}_{it}\right)\mu_{it}, \end{array}$$

In equation (9), the probability expression *p*(γ_*i*_+ρŹ_*it*_>0), is equal to one, whereas α, β, γ, and ρ represent the values that this study chooses to forecast. The subscript (*i*) denotes the fixed effect described by the parameters α_*i*_ and γ_*i*_, which would be constrained to the values *i* = 1, 2, …, *n*. Consequently, the distinctive component of *R*, denoted by *Z*, is the *k*-vector, while the vector denotes the variation“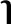.”


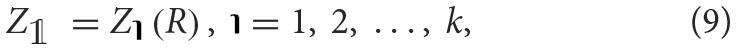


In the preceding equation, *R*_*it*_ is distributed independently and identically for the overall fixed *i* and time (*t*), which is orthogonal to both *i* and *t* (50). Consequently, the outside elements and reserves are both stable. Based on the above argument, both research models [Eq. (1) and Eq. (2)] may be reformulated as follows:


(10) $$\begin{array}{l} Q_{y}\left(\tau |R_{it}\right)=\left(\alpha_{i}+\gamma_{i}q\left(\tau \right)\right)+\beta R_{it}+\rho {Ź}_{it}q\left(\tau \right), \end{array}$$

In this transformed research model, the set of explanatory variables, which comprises *HHW, BQ, URP, GDPPC, and EPR* (Model-1), while the *HHW, DA, URP, GDPPC, EPR* (Model-2) variables, all of these variables are captured by *R*_*it*_. Besides, all of these variables are converted into natural logarithms, rendering them unitless to express the estimated outcomes as a percentage. Furthermore, *R*_*it*_ reflects the quantile distribution of the dependent variable, as shown by *Y*_*it*_ and is supposed to be *DGHE* in this case, which also depends on the quantile location. Moreover, the expression −α_*i*_(τ) ≡α_*i*_+γ_*i*_*q*(τ) Reflects the scalar portion that generates the fixed impact of τ quantiles on *i* nevertheless, these quantiles have no consequence on the intercept. Certain outputs are susceptible to change due to the factors' structural independence. Lastly, *q*(τ) represents the τ−*th* quantile sample, which are Q_0.25_, Q_0.50_, Q_0.75_, and Q_0.90_ in this study. Therefore, the quantile equation used in this study is as follows:


(11) $$\begin{array}{l} min_{q}\sum_{i}\;\sum_{t}\theta_{\tau }\left(R_{it}-\;\left(\gamma_{i}+\;\rho {Ź}_{it}\right)q\right) \end{array}$$

Where θ_τ_(*A*) = (τ−1) *AI*{*A* ≤ 0}+*TAI*{*A*>0} stands for the testing function.

Although the empirical results of the MMQR provide estimated results at a specific location and scale, this study still verifies the robustness of the long-run estimators. Therefore, the non-parametric panel approach, i.e., quantile regression proposed by Koenker and Bassett Jr. (49), is used to assess the influence of each explanatory variable on *DGHE*. Besides, the quantile regression will also provide evidence for the validity of the models under consideration. In addition to identifying the long-run estimator, this research aims to analyze the causative link between CO2 emissions and the regressors since previous estimating methods did not demonstrate a causative link between the studied variables. In this context, the present work employs the pairwise panel Granger causality test developed by Dumitrescu and Hurlin (52), which is more effective at addressing the mentioned problems with panel data.

## Results and discussions

The results and their interpretation is elaborated in this segment of the study. The study utilizes cointegration analysis, Quantile regressions, and causality tests to examine the variables' association.

### Pre-estimation diagnostics

The descriptive statistics and normality results are reported in Table 1. The average values are expressed by mean and median values, which are nearly close to each other. The standard deviation shows the deviation from mean values or data spread of the variables. Moreover, the skewness and Kurtosis values lie in the range as illustrated by (53), depicting the distribution and peakedness of the variables. In general, the data is non-normal with skewed distribution.

**Table 1:** Descriptive statistics and normality check.

	**DGHE**	**HHW**	**BQ**	**DA**	**URP**	**GDPPC**	**EPR**
Mean	7.985048	10.42534	3.882533	5.677267	18.05553	10.58271	23.93915
Median	8.071815	10.32840	4.000000	6.000000	17.88389	10.54866	23.77579
Maximum	8.622022	11.84898	5.000000	6.000000	19.42876	11.01349	26.48349
Minimum	7.314934	8.857928	3.000000	4.000000	16.95454	10.31094	21.55269
Std. Dev.	0.294028	0.715293	0.348899	0.466082	0.702314	0.172524	1.274544
Skewness	−0.370871	0.617970	−1.425295	−1.021327	0.547105	0.588474	0.099974
Kurtosis	2.494274	3.168797	6.198084	2.732857	2.399756	2.533737	2.157220
Jarque-Bera	5.037124	9.725252	114.7101	26.52377	9.734920	10.01629	4.689110
Probability	0.080575	0.007730	0.000000	0.000002	0.007693	0.006683	0.095890
Observations	150	150	150	150	150	150	150

### Slope heterogeneity and cross-section dependence

Attributable to the presence of financial, socio-economical, and technical factors among a different set of countries under the cross-section might prevail some comparisons and divergences, or else the presence of some unknown factors affects the variables. These sometimes tend to give biased outcomes. Therefore, the study employs Slope heterogeneity and Cross-sectional dependence for reliable and efficient results.

Table 2 presents the homogeneous or heterogeneous slope results of both models. The statistical values of both models (1&2) reject the null hypothesis of homogeneity at a 1% significance level. The findings demonstrate that both models are heterogeneous, leading to the analysis of cross-sectional dependence. Table 3 shows the results of the cross-sectional dependence of the variables under study by Pesaran (54). The statistical values present that the variables reject the null hypothesis of no inter-dependence. The variable is significant at 1 and 10% levels of significance across the panel, illustrating that variables are correlated and interdependent in the panel.

**Table 2:** Slope heterogeneity.

**Homogenous/heterogeneous slope coefficient testing**	
$\tilde{\Delta }$	${\tilde{\Delta }}^{Ajusted}$
**Model-1**
3.261***	3.843***
**Model-2**
4.358***	5.136***

Asterisks indicate a statistical significance level of 1% (***), 5% (**), and 10% (*).

**Table 3:** Cross-section dependence test.

**Pesaran** **(****54****)** **CD Test**		
**Variable**	**CD-test**	**Corr**
DGHE	14.40***	0.743
HHW	1.75*	0.090
DA	1.74*	0.090
URP	17.93***	0.926
GDPPC	18.19***	0.939
EPR	13.78***	0.711

Asterisks indicate a statistical significance level of 1% (***), 5% (**), and 10% (*).

### Unit root analysis

The discoveries of cross-sectional dependence lead toward examining unit root analysis of the variables. Transforming the conventional ADF test, the study utilizes Pesaran Unit root analysis (46) and the estimated result for this test is provided in Table 4. The variables such as Domestic government health expenditures (DGHE), Democratic accountability (DA), and urban growth (URP) provide significant results at level I(0), giving the order of integration I(0). At the same time, all other variables, Household waste (HHW), Bureaucratic quality (BQ), economic growth (GDPPC), and electricity production from renewables (EPR), give significant results at integration order I(1) and reject the hypothesis at the first difference on a 1% level of significance. The coefficients having a negative sign indicate the presence of a unit root, i.e., the greater the negative values specify, the higher will be the unit root.

**Table 4:** Unit root testing (46).

**Variable(s)**	**Trend and intercept**		**Order of integration**
	**I(0)**	**I(1)**	
DGHE	−3.289***	-	I(0)
HHW	−2.542	−4.098***	I(1)
BQ	−0.832	−2.910**	I(1)
DA	−2.748*	-	I(0)
URP	−2.799*	-	I(0)
GDPPC	−2.162	−3.359***	I(1)
EPR	−1.941	−4.090***	I(1)

Asterisks indicate a statistical significance level of 1% (***), 5% (**), and 10% (*).

### Cointegration analysis

Detecting unit root among the variables leads us to estimate the long-run relationships or con-integration analysis. The study uses Kao Cointegration, and Pedroni Cointegration tests to assess the association. The analysis consists of mentioned tests in Table 5, where the null hypothesis of cointegration analysis exhibits no cointegration among variables. The statistical values in both model 1 and model 2 demonstrate the existence of cointegration, rejecting the null hypothesis.

**Table 5:** Cointegration test.

**Test**	**Statistic (Model-1)**	**Statistic (Model-2)**
**Kao cointegration test**
Modified DF t	−5.3388***	−4.1087***
DF t	−3.8503***	−3.5916***
ADF t	−4.8057***	−4.9346***
Unadjusted modified DF t	−5.3181***	−4.9900***
Unadjusted DF t	−3.8462***	−3.8129***
**Pedroni cointegration test**
Modified PP t	2.4069***	2.8419**
PP t	0.7679	1.4226*
ADF t	0.4317	0.7302

DF is Dickey-Fuller, ADF represents Augmented Dickey-Fuller, and PP reports Phillips-Perron. Asterisks indicate a statistical significance level of 1% (***), 5% (**), and 10% (*).

The overall results illustrate that the variable has a long-run relationship. Bureaucratic Quality and Democratic Accountability, Economic Growth, Renewable Energy use, Household waste, and Urban population have a significant long-run relationship with Government Health expenditures. The statistical values present the momentous association by Modified Dickey-Fuller, Dickey-Fuller, ADF, Unadjusted modified DF, Unadjusted DF, and Pedroni Cointegration test.

### Long-run results

When linearity and residual normality conditions are not satisfied, Quantile Regressions are applied to estimate the relationships among the variables. Tables 6, 7 of the results section demonstrate the findings from Quantile regressions with each model graphical representation correspondingly. Tables 8, 9 show the robustness test outcomes with graphical analysis, respectively.

**Table 6:** Estimates of quantile regression–MMQR (Model-1).

**Variable**	**Location**	**Scale**	**Quantiles**			
			**Q_0.25_**	**Q_0.50_**	**Q_0.75_**	**Q_0.90_**
HHW	−0.170*** [0.054]	−0.015 [0.032]	−0.158*** [0.057]	−0.171*** [0.054]	−0.187*** [0.066]	−0.200** [0.086]
BQ	−0.156** [0.063]	0.088** [0.037]	−0.225*** [0.067]	−0.153** [0.064]	−0.065 [0.079]	0.010 [0.101]
URP	0.054 [0.054]	0.076** [0.032]	−0.004 [0.057]	0.057 [0.055]	0.132* [0.067]	0.198** [0.087]
GDPPC	0.740*** [0.157]	0.008 [0.094]	0.734*** [0.165]	0.741*** [0.157]	0.749*** [0.193]	0.756*** [0.249]
EPR	0.115*** [0.026]	−0.068*** [0.015]	0.168*** [0.290]	0.112*** [0.028]	0.045 [0.034]	−0.014 [0.043]
Constant	−1.208 [1.390]	0.153 [0.831]	−1.326 [1.463]	−1.202 [1.394]	−1.051 [1.711]	−0.919 [2.209]
**Graphical Representation of MMQR - Model-1**						
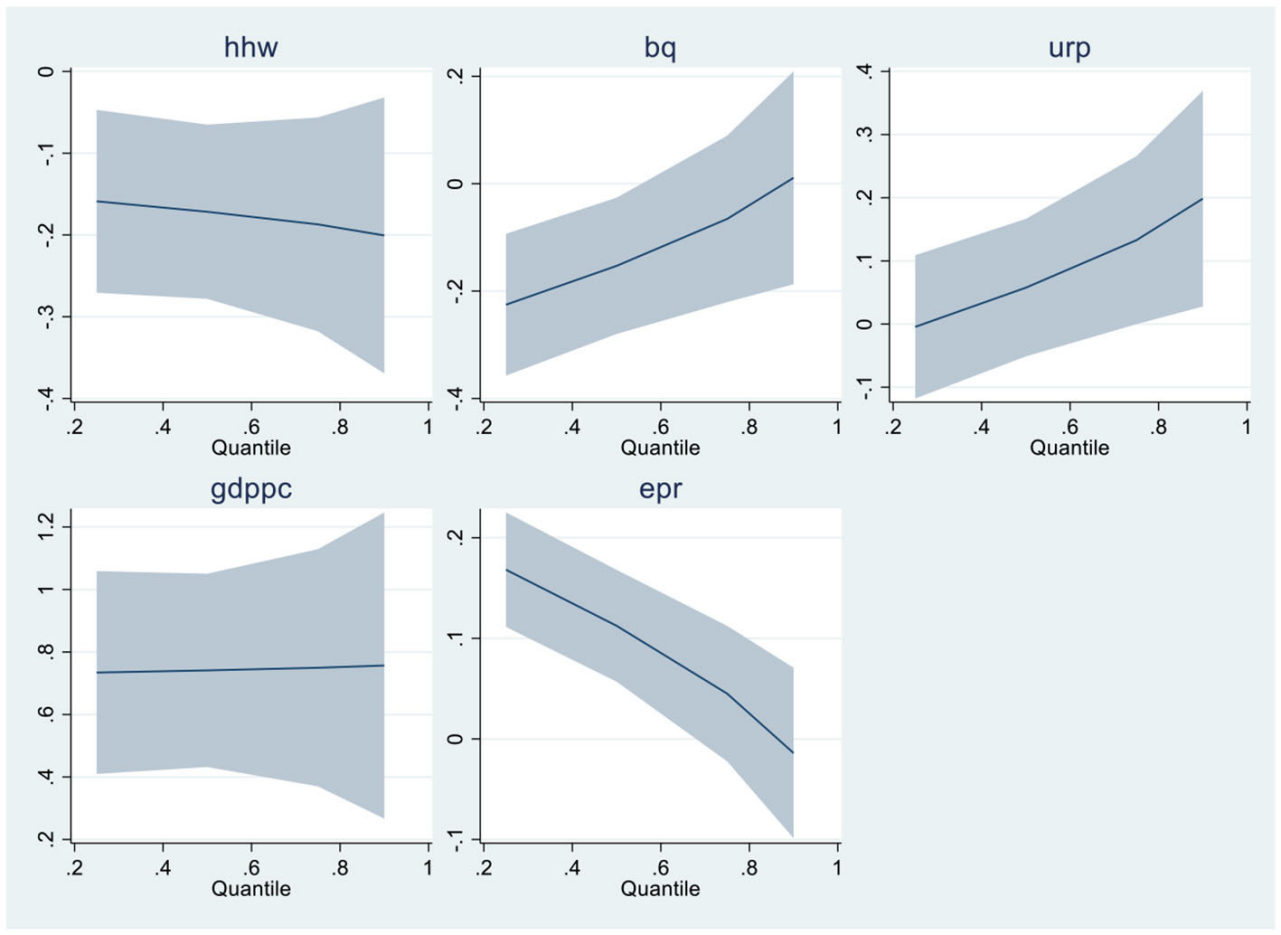						

The dependent variable is DGHE. Asterisks indicate a statistical significance level of 1% (***), 5% (**), and 10% (*).

**Table 7:** Estimates of quantile regression–MMQR (Model-2).

**Variable**	**Location**	**Scale**	**Quantiles**			
			**Q_0.25_**	**Q_0.50_**	**Q_0.75_**	**Q_0.90_**
HHW	−0.143*** [0.047]	0.006 [0.027]	−0.149*** [0.052]	−0.143*** [0.047]	−0.136** [0.055]	−0.132** [0.065]
DA	−0.026 [0.058]	0.026 [0.033]	−0.048 [0.064]	−0.026 [0.058]	0.001 [0.068]	0.016 [0.080]
URP	0.025 [0.057]	0.067** [0.032]	−0.029 [0.063]	0.027 [0.058]	0.096 [0.067]	0.135* [0.079]
GDPPC	0.715*** [0.167]	−0.097 [0.095]	0.795*** [0.184]	0.713*** [0.168]	0.613*** [0.196]	0.557** [0.230]
EPR	0.111*** [0.024]	−0.063*** [0.013]	0.163*** [0.027]	0.109*** [0.026]	0.045 [0.029]	0.008 [0.035]
Constant	−1.068 [1.274]	1.273* [0.724]	−2.100 [1.400]	−1.033 [1.286]	0.260 [1.493]	0.999 [1.758]
**Graphical Representation of MMQR - Model-2**						
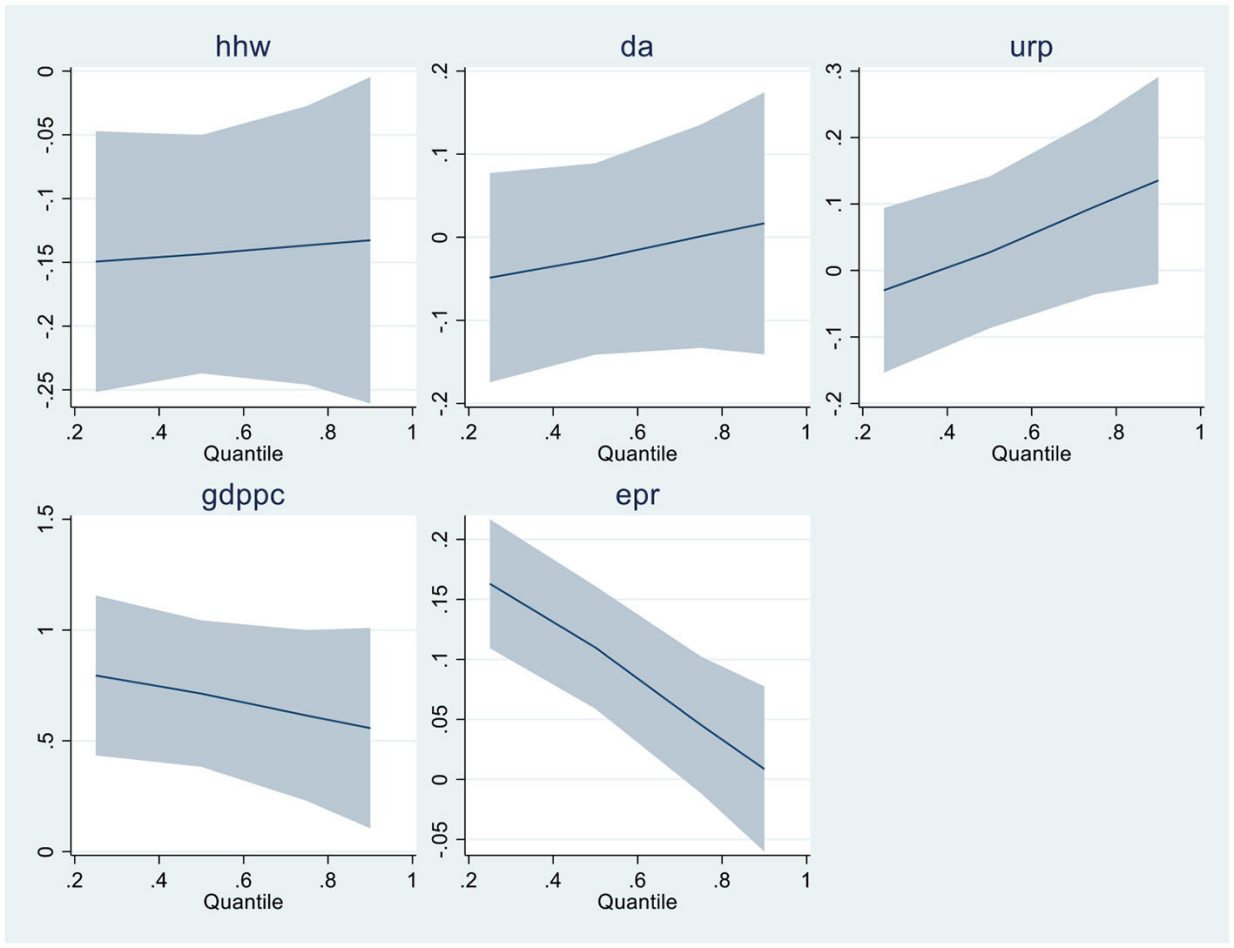						

The dependent variable is DGHE. Asterisks indicate a statistical significance level of 1% (***), 5% (**), and 10% (*), while the standard error is provided in Brackets.

**Table 8:** Robustness test—quantile regression – (Model-1).

**Variable**	**Quantiles**			
	**Q_0.25_**	**Q_0.50_**	**Q_0.75_**	**Q_0.90_**
HHW	−0.155**	−0.178**	−0.140	−0.033
BQ	−0.300***	−0.223***	−0.112	0.023
URP	−0.028	0.014	0.068	0.070
GDPPC	0.411**	0.805***	0.654***	0.549***
EPR	0.236***	0.154***	0.084**	0.016
Constant	1.154	−1.792	−0.142	1.015
**Graphical Representation of Quantile Regression - Model-1**				
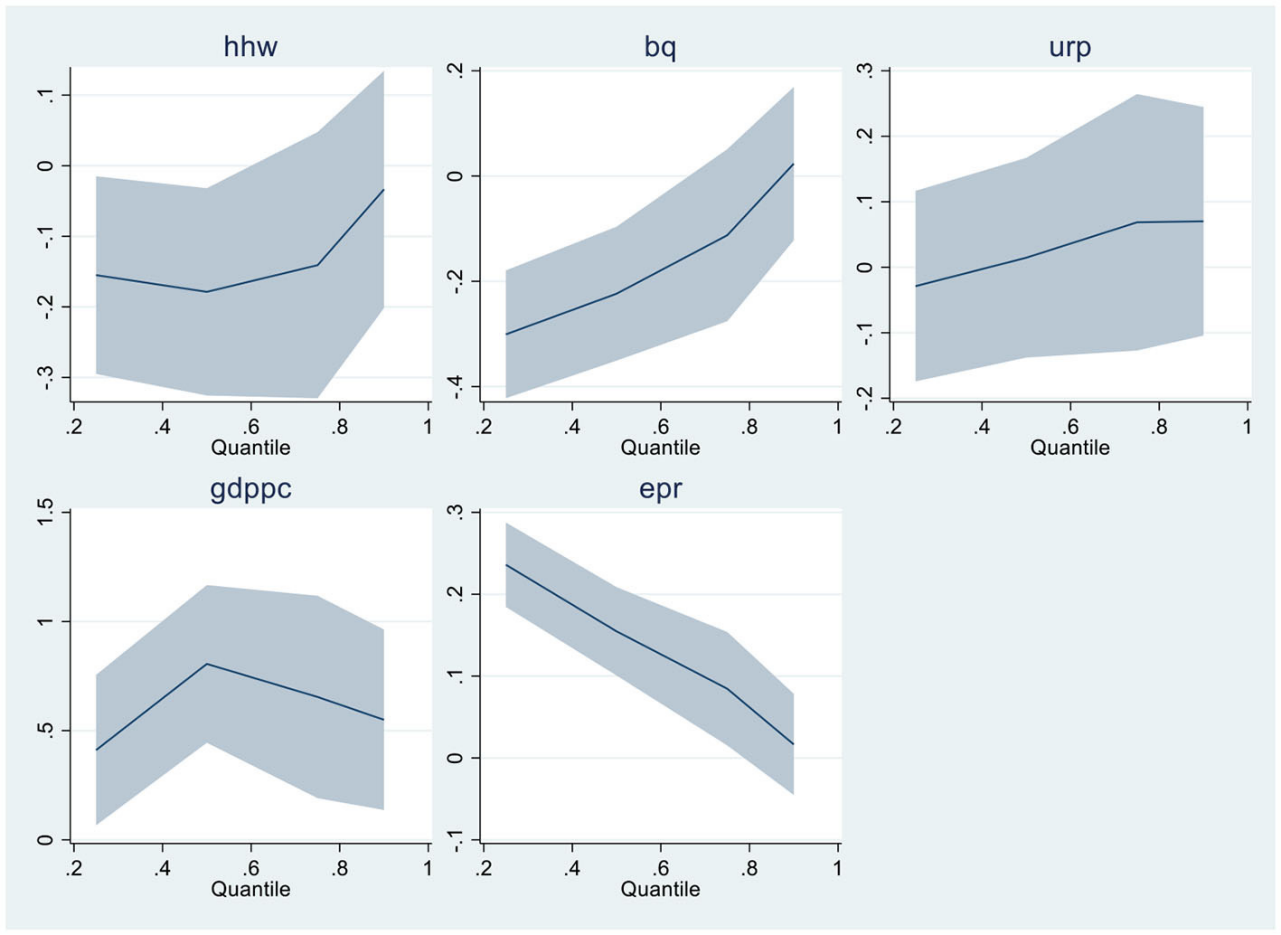				

The dependent variable is DGHE. Asterisks indicate a statistical significance level of 1% (***), 5% (**), and 10% (*).

**Table 9:** Robustness test—quantile regression – (Model-2).

**Variable**	**Quantiles**			
	**Q_0.25_**	**Q_0.50_**	**Q_0.75_**	**Q_0.90_**
HHW	−0.136*	−0.097	−0.079	−0.031
DA	−0.048	−0.018	−0.018	−0.074
URP	−0.068	−0.044	0.044	0.074
GDPPC	0.507**	0.759***	0.665***	0.501**
EPR	0.228***	0.13^1***^	0.047	0.032
Constant	−0.060	−1.274	0.126	1.575
**Graphical Representation of Quantile Regression - Model-2**				
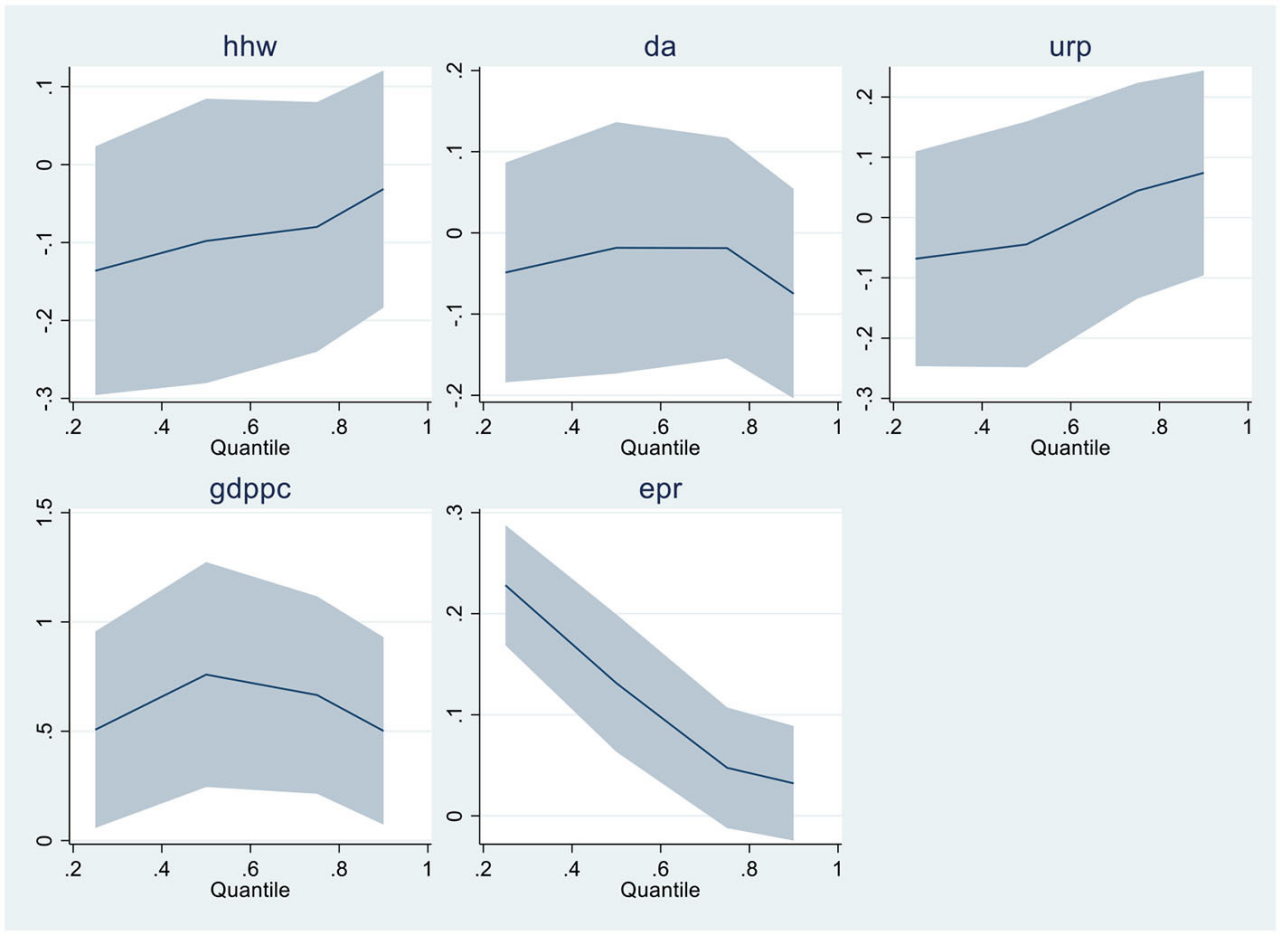				

The dependent variable is DGHE. Asterisks indicate a statistical significance level of 1% (***), 5% (**), and 10% (*).

The Model 1 results are in Table 6; the bureaucratic quality is negatively and significantly associated with health expenditures. The upsurge and improvement in bureaucratic quality increase the government health expenditures in the country. The result is consistent with Makuta and O'Hare (37) that improved quality of bureaucracy reduces improved health outcomes by increasing expenditures by the government. Urban population, economic growth, and renewable electricity production are positively associated with health expenditures in G6 economies. The findings are consistent with succeeding studies. A percentage increase in these variables leads to increased expenditures in the economy. Economic activity increases pollution and health expenses (16). Globalization increases the urban population, which positively impacts health expenditures (28). The impact of renewable energy on health expenditures is in-line with Sasmaz et al. (21). Because as the consumption of renewable energy increases, the funds for health expenses increase by the government eventually used for development resolutions. Household waste is negatively associated with health expenditures in G6 economies. The finding is substantial and in line with (25) that household waste impact health expenses. The study revealed significant factors that influence health expenditures. The graphical presentation of all quantiles from model 1 is presented with MMQR model 1.

In model 2, democratic accountability is positively associated with health expenditures in upper quantiles while harmful in lower quantiles. The result is consistent somehow with Liang and Mirelman (34) that the improvement in governance accountability tends to increase health expenditures in an economy when the economy is stable.

Similarly, as stated in model 1, a percentage increase in URP, GDPPC, and EPR variables leads to increased health expenses. Increasing economic activity increases pollution, which tends to increase health expenses (16). Globalization increases the urban population, which positively impacts health expenditures (28). The impact of renewable energy on health expenditures is in-line with Sasmaz et al. (21). Because as the consumption of renewable energy increases, the funds for health expenses increase by the government eventually used for development resolutions. The graphical representation of model 2 is presented in MMQR model 2.

### Robustness tests and causality analysis

After the fresh findings in MMQR analysis, the robustness analysis is applied using Quantile regressions. Economic growth, household waste, and electricity from renewable sources depicted significant results in both models 1 and 2. At the same time, Bureaucratic quality presented negative and substantial results in upper quantiles. The bureaucratic accountability depicts negative but insignificant results in the robustness analysis. The graphical presentation is illustrated accordingly.

Lastly, causality analysis is applied to examine the variables' causal association the results are presented in Table 10. Five pairs of twelve variables have shown significant results and rejected the null hypothesis of no causality. Household waste substantially causes government health expenditures in G6 nations. Bureaucratic quality has a causal association with government health expenditures. Democratic accountability significantly causes unidirectional association with health expenditures. Similarly, economic growth and renewable energy have a significant causal relationship with health expenditures. All these pairs have shown a unidirectional association with government health expenditures.

**Table 10:** Causality test.

**Pairwise granger causality test**		
**Null hypothesis**	**F-statistics**	**Prob**
HHW does not granger cause DGHE	2.63764**	0.0374
DGHE does not granger cause HHW	1.61838	0.1742
BQ does not granger cause DGHE	2.54611**	0.0431
DGHE does not granger cause BQ	1.86006	0.1221
DA does not granger cause DGHE	8.87541***	3.E-06
DGHE does not granger cause DA	0.45768	0.7666
URP does not granger cause DGHE	0.65654	0.6234
DGHE does not granger cause URP	0.39741	0.8102
GDPPC does not granger cause DGHE	4.86567***	0.0012
DGHE does not granger cause GDPPC	1.04780	0.3857
EPR does not granger cause DGHE	3.37103***	0.0119
DGHE does not granger cause EPR	0.37891	0.8233

Asterisks indicate a statistical significance level of 1% (***), 5% (**), and 10% (*).

### Empirics discussion

The above section provided the estimated findings from the econometric analysis for evaluating the role of Household waste, Governance quality, and Greener Energy for Public Health expenditures. The descriptive statistics of the research information presented a non-normal and skewed distribution which leads to the analysis of Panel Quantile regressions. At first, the unit root analysis depicted a strong presence of integration. Then the cointegration analysis portrayed that variables have long-run associations. The momentous associations are presented by Kao cointegration and Pedroni cointegration tests. Then Quantile regressions demonstrated the direction of associations between dependent and explanatory variables. The variable under consideration such as Urban growth, Gross Domestic Product, and electricity from renewable energy sources showed consistent findings with studies (16, 21, 28). Moreover, the association of democratic accountability is nearly in-line with Liang and Mirelman (34) that the improvement in governance accountability tends to increase health expenditures. In comparison, the bureaucratic quality has been shown by some means consistent outcomes with Makuta and O'Hare (37), revealing that enhanced quality of bureaucracy tends to decrease health risks by increasing expenditures by the government. Lastly, the causal association analysis showed a one-way causal relationship between bureaucratic accountability, democratic quality, renewable energy, and household waste significantly impacting health expenditures. The causality analysis is applied to examine the direction of causal association flowing from dependent to independent variable or vice versa.

The overall findings demonstrated that the increasing environmental degradation by harmful pollution from non-renewable energy consumption leads to increasing health concerns. Besides, household waste, pollution, and poor governance quality have also contributed to reducing people's health standards. The harmful waste not only sabotages the environmental quality but also affects public health by developing harmful or deathly diseases in humans. These health risks have a substantial and detrimental impact on government health spending. The government intervention in the health sector and managing its funds for improvement in sustainable development must be focused on for better health of the public. For that reason, the provision of health facilities is the responsibility of the government and they are accountable to their people. Therefore, an effective and efficient democratic regime and accountability can be resourceful in enhancing health expenses for the public. Alongside, greener energy is essential for improving the environmental and health quality of individuals. Hence, the study adds to the debate on health academics economically and environmentally by providing substantial empirical evidence.

## Conclusion and policy implications

Utilizing the updated data sample from 1996 to 2020, the study analyzes the association between governance, Economic Growth, Electricity from Renewable Energy sources, Household waste, and Urban population on Government Health expenditures in G6 countries. It is not entirely astonishing that the said variables substantially impact health expenditures. However, deviating from existing studies, the authors utilize Bureaucratic Quality and Democratic Accountability as proxies for governance and their influence on government health expenses in developed economies for the first time concurrently. Further, the impact of household waste on health expenditures is also significant in the findings.

The findings of the study are novel and substantial in policy making. Nevertheless, in the existing literature, a few studies have examined the factors in the health sector. The following sets of studies are in-line with prevalent literature (16, 21, 25, 28, 34, 37). The government's involvement in the health sector spending and investment, and also managing its funds for improvement in sustainable development, must be focused on for better public health.

Based on the results, the following are the policy implications.

The availability of health funds to countries must be improved because it supports providing fundamental healthcare facilities to the citizens. The basic fundamentality of health is the responsibility of the local governments. Therefore, democratic quality and bureaucratic accountability efficiency are substantial for health sustainability. Efficient and effective governance is needed to achieve health targets, and efficient strategies are required to be implemented for better health sustainability.

To secure the world from global health challenges, the Group of Seven economies must come forward to ensure health sustainability. These countries have a substantial role in revamping strategies by making an example for other countries worldwide. The G6 members must consolidate to organize R&D for healthcare improvement, increase health investment, and fund vulnerable economies. The developed economies have a major role in shaping global policies for sustainability. Supplementary, they have acquired relevant policies and accountability practices, tackling countless health crises and commitments to encouraging sustainability. Lately they joined global submit to mitigate health concerns to fortify strategies for health tragedies (5, 6).

These G6 developed economies must invest in green innovation for a cleaner environment. The countries must encourage the healthcare systems and healthcare workforce. Consequently, the study has significantly examined the role of governance and clean energy on developed nations' public health to provide outcomes that are considered in evaluating policies for mitigating health challenges.

Better waste management is needed for vulnerable areas because it deteriorates health and environmental quality. In addition, it strengthens the disease response preparedness for future purposes. Therefore, the five R's of waste management is an essential step toward improving health and environmental sustainability. Recycle, refuse, reduce, repurpose and reuse the waste which is cost-effective and environmentally feasible. These will aid in minimizing household waste generation. Additionally, proper roles and responsibilities besides the policies of each activity in the waste management chain must be strategized and re-vamped under Global Environmental Protection Act.

### Limitations and recommendations

Despite the importance of research, the study can be replicated in other countries with more available data because the study is limited to G6 economies due to data restrictions. Future researchers can also expand the research by including similar or assessing other governance variables on public health-reflecting variables such as institutional quality, financial inclusion, child and adult mortality rates, education expenditures, R&D development, etc.

## Data availability statement

The original contributions presented in the study are included in the article/supplementary material, further inquiries can be directed to the corresponding author.

## Author contributions

JW: concept, data, software, and analysis. LX: analysis, methods, preparing draft, and literature review. JZ: results, discussion, theory, concept, and implications. All authors contributed to the article and approved the submitted version.

## Conflict of interest

The authors declare that the research was conducted in the absence of any commercial or financial relationships that could be construed as a potential conflict of interest.

## Publisher's note

All claims expressed in this article are solely those of the authors and do not necessarily represent those of their affiliated organizations, or those of the publisher, the editors and the reviewers. Any product that may be evaluated in this article, or claim that may be made by its manufacturer, is not guaranteed or endorsed by the publisher.

## Footnotes

^1^For data and information, visit: https://databank.worldbank.org/source/world-development-indicators.
